# Development of B Cell Memory in Malaria

**DOI:** 10.3389/fimmu.2019.00559

**Published:** 2019-04-02

**Authors:** Ann Ly, Diana S. Hansen

**Affiliations:** ^1^The Walter and Eliza Hall Institute of Medical Research, Parkville, VIC, Australia; ^2^Department of Medical Biology, The University of Melbourne, Parkville, VIC, Australia

**Keywords:** malaria, immunity, antibodies, memory B cells, inflammation

## Abstract

A single exposure to many viral and bacterial pathogens typically induces life-long immunity, however, the development of the protective immunity to *Plasmodium* parasites is strikingly less efficient and achieves only partial protection, with adults residing in endemic areas often experiencing asymptomatic infections. Although naturally acquired immunity to malaria requires both cell-mediated and humoral immune responses, antibodies govern the control of malarial disease caused by the blood-stage form of the parasites. A large body of epidemiological evidence described that antibodies to *Plasmodium* antigens are inefficiently generated and rapidly lost without continued parasite exposure, suggesting that malaria is accompanied by defects in the development of immunological B cell memory. This topic has been of focus of recent studies of malaria infection in humans and mice. This review examines the main findings to date on the processes that modulate the acquisition of memory B cell responses to malaria, and highlights the importance of closing outstanding gaps of knowledge in the field for the rational design of next generation therapeutics against malaria.

## B Cell Immunological Memory

**Figure 1:**
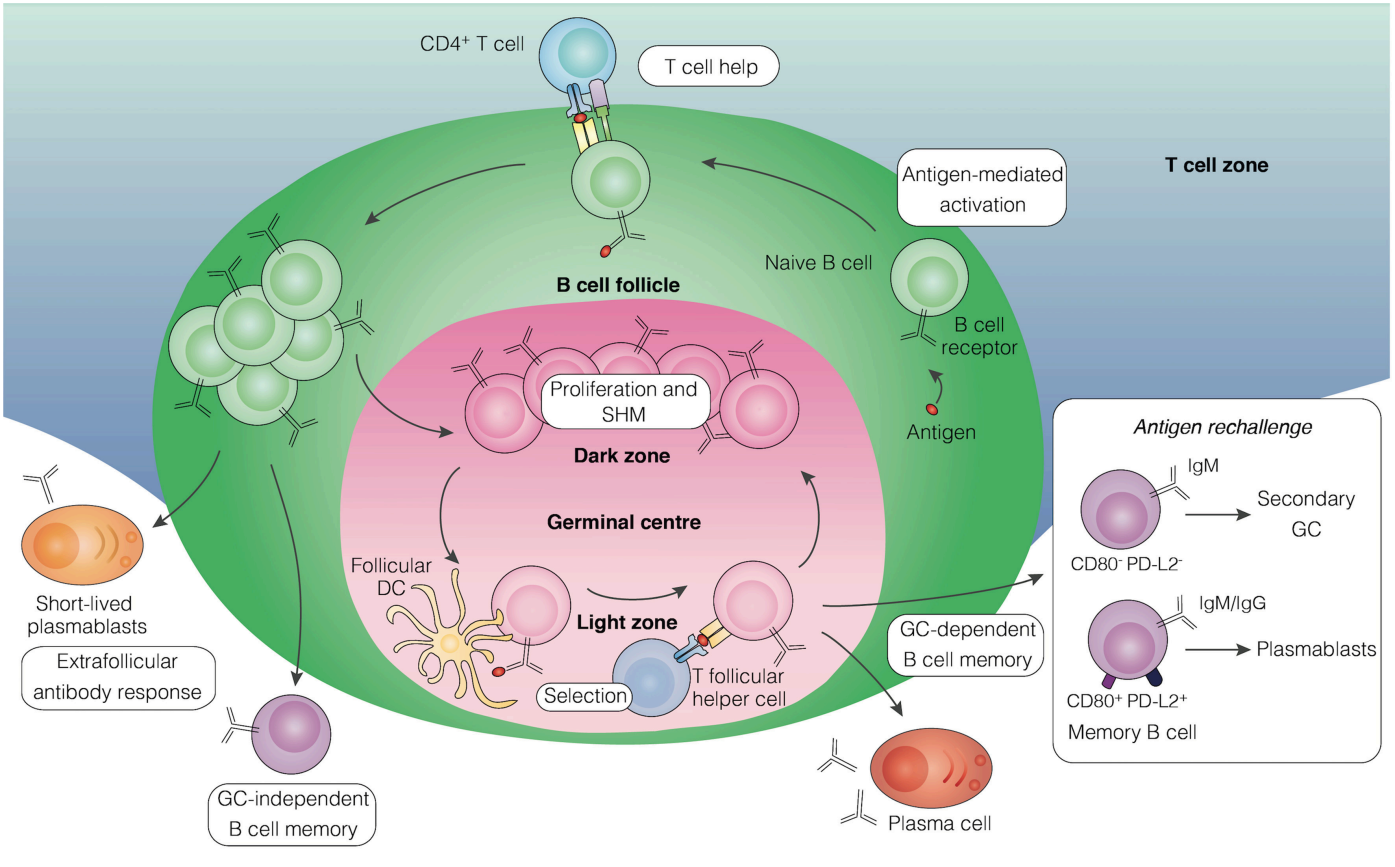
Pathways leading to the development of B cell memory. Upon encounter with antigen, activated B cells in secondary lymphoid tissue receive helper signals from cognate CD4^+^ T cells at the border of the B cell follicle and T cell zones. Some of the proliferating B cells differentiate into short-lived plasmablasts that initiate an extrafollicular antibody response, some develop into early memory B cells independently of GC formation, while others aggregate into the follicle to establish a GC. Within the GC, B cells undergo proliferation and somatic hypermutation in the dark zone, followed by affinity-based selection in the light zone with the help of T follicular helper cells and follicular dendritic cells. Long-lived plasma cells and memory B cells emerge from the GC reaction. Upon antigen rechallenge, memory B cells lacking expression of the surface molecules CD80 and PD-L2, mainly of the IgM isotype, can seed secondary GCs, whereas those expressing both molecules, comprising of IgM and IgG isotypes, predominantly generate plasmablasts. GC, germinal center; DC, dendritic cell; SHM, somatic hypermutation.

Immunological memory refers to the ability of the vertebrate immune system to remember previously encountered antigens or pathogens and evoke an enhanced immune response to control infection. The capacity of the host to generate T and B cell memory underlies the basis of protective immunity induced by vaccination or after exposure to specific pathogens. The generation of T cell-dependent humoral immune memory in secondary lymphoid organs (Figure 1) typically begins following B cell engagement with its cognate antigen, which triggers their migration to the B cell follicle border to receive T cell help (1). Activated B cells then differentiate along one of three possible routes, leading to the rapid production of short-lived plasmablasts, generating germinal center (GC)-independent memory B cells (MBCs), or formation of GCs in B cell follicles (2, 3). GCs establish within a few days of initial antigen encounter and mature into two distinct micro-anatomical compartments: the dark zone, where B cell clones undergo proliferative expansion and somatic hypermutation of their immunoglobulin (Ig) genes, and the light zone, where B cells expressing high-affinity antibodies are selected and undergo class switch recombination (4–6). The GC reaction leads to the generation of affinity-matured MBCs and long-lived plasma cells that contribute to host protection against re-infection. Plasma cells migrate to the bone marrow and provide a continuous source of circulating high-affinity antibody (7), while MBCs recirculate in the blood and secondary lymphoid tissue (8) to induce a rapid effector response upon antigen re-encounter (9, 10).

Comprehensive studies of MBC biology have led to the appreciation of substantial heterogeneity among the MBC compartment, consisting of distinct subpopulations with different effector capacity upon secondary challenge (11). In humans, the expression of unique memory-specific surface markers has been extensively used to identify and characterize MBCs. Surface expression of CD27 defines a subset of antigen-experienced MBCs in humans that are class-switched and bear Ig variable region mutations (12, 13). However, CD27 expression does not universally define all MBCs, as subsequent work identified an CD27^−^ CD21^−^ MBC population (14). These cells, coined as atypical MBCs in malaria, express several Fc receptor-like (FcRL) inhibitory receptors, including FcRL3 and 5 (15–17).

The development of novel labeling techniques to track antigen-specific B cells in murine models has enabled the functional characterization of MBCs expressing different antibody isotypes. Whereas, IgG MBCs have been found to preferentially differentiate into plasmablasts upon antigen rechallenge, IgM MBCs appear to have the capacity to re-enter secondary GC reactions but were not enhanced in plasmablast generation (18, 19). Recent studies have uncovered additional heterogeneity within the IgM and IgG MBC pools, proposing that MBCs lacking the memory markers CD80 and PD-L2, which primarily express IgM, are dedicated to reseeding GCs, while those expressing both markers, comprising of IgM and IgG isotypes, contribute mainly to immediate antibody-secreting function (Figure 1) (20). Consistently, IgM-expressing MBCs have recently been shown to exhibit considerable plasticity in their differentiation capacity following rechallenge (21). This division of labor in the MBC response has been proposed to support a rapid and effective response upon antigen rechallenge while concurrently permit the generation of new MBCs (11). How these findings in murine models relate to human settings is unclear, as there is still little information about the homology between mouse and humans MBC subsets with regards to their surface marker expression usage.

## A Role for Memory B Cels in Naturally Acquired Immunity to Malaria

The global burden of malaria has more than halved since the turn of the century due to renewed eradication efforts, but progress has recently stalled as current intervention strategies are confronted with several major challenges, including the emergence of anti-malarial drug and insecticide resistance (22). Of the six *Plasmodium* species known to infect humans, *P. falciparum* continues to account for the majority of deaths, whereas recurrent *P. vivax* episodes are a significant source of morbidities. Disease syndromes of malaria range from fever to more severe complications including acute pulmonary oedema, jaundice, severe anemia, hypoglycaemia, acidosis, and cerebral malaria (23). The pathogenesis of malarial disease is thought to arise from the concerted effects of host and parasite mechanisms, including the sequestration of blood-stage parasites in microvasculature, and local and systemic inflammation induced by the parasites and their toxic products (24, 25).

Early epidemiological observations by Robert Koch in malaria-endemic populations described that natural immunity to malaria can be achieved, but requires years of repeated exposure to *Plasmodium* parasites (26). Children living in high transmission regions become immune to the most severe forms of malaria after relatively few symptomatic infections (27–29), but remain at risk of uncomplicated malaria. After years of repeated infections with age, protection from successive malaria episodes or “clinical immunity,” is acquired by the ability to substantially reduce parasite burdens (30–35). This form of protection is not paralleled by sterile immunity that prevents re-infection (36), and adults continue to be experience low-density, asymptomatic infections throughout life (37). Naturally acquired clinical immunity to malaria targets blood-stage parasites and requires antibodies, as demonstrated by studies in which the transfer of purified IgG from malaria-immune adults to children with symptomatic malaria rapidly reduced parasitemia and fever (38). Together, these observations have led to the hypothesis that the slow and imperfect acquisition of immunity to malaria reflects in the development of MBCs, and this topic has been the subject of several studies including mouse infection models as well as human settings. Here we review our current understanding on the salient features of the development of humoral immunity to malaria infection, and highlight some of the outstanding questions regarding the cellular mechanisms that underlie the slow acquisition of clinical immunity.

## Antibody Responses to Blood-Stage Malaria

The paramount importance of antibodies in controlling blood-stage malaria infection was proven by seminal passive-transfer experiments, in which IgG from *P. falciparum* clinically immune adults protected non-immune children from high parasitemia and clinical symptoms (38, 39). Numerous immuno-epidemiological studies subsequently demonstrated that high antibody levels against specific blood-stage parasite antigens correlate with protection from disease (40–46). Antibodies may control the development of clinical symptoms by targeting the invasion and growth of the merozoite form of the blood-stage parasite and redirect their clearance by phagocytic cells via Fc and complement receptors (47). Additionally, antibodies directed against parasite antigens expressed on infected erythrocytes can promote opsonic phagocytosis, block microvasculature adherence, disrupt rosette formation with uninfected cells, and prevent erythrocyte rupture and parasite egress (47).

Antibodies may target a number of highly polymorphic and functionally redundant antigens expressed by *Plasmodium* parasites (48), which may represent a potential mechanism by which the parasite effectively evades the human immune system via antigenic variation (49). Asymptomatically-infected individuals who fail to mount an antibody response against *P. falciparum* has been shown to predict increased susceptibility to clinical disease (50, 51). In parallel, individuals detected with multi-clonal *P. falciparum* infections in the dry season have been associated with subsequent protection from febrile malaria (52), suggesting that the presence of persisting parasites enhance antibody recognition and enable cross-reactive responses. This supports the notion that clinical immunity may depend on the cumulative acquisition of a repertoire of antibodies to a diverse range of parasite antigens or development of cross-species antibody responses (53–55). Indeed, the breadth of parasite-specific antibody responses have been identified to increase with age in endemic populations (56–58), and the antibody repertoire diversifies rapidly during infancy but plateaus in toddlers (59). Moreover, antibodies with broad reactivity against *P. falciparum* that carry a gene insertion derived from the collagen-binding protein LAIR1, have been shown to undergo somatic hypermutation that increase binding to infected erythrocytes (60). LAIR1 insertions have been further revealed to represent a relatively common mechanism of antibody diversification in African individuals, and that broadly-neutralizing antibodies against *Plasmodium* arise from these low-affinity precursors over time (61). While antigenic variation has been proposed to explain the slow acquisition immunity to malaria, there is also an increasing body of evidence suggesting that antibody responses to malaria are poorly generated. In malaria-endemic areas, substantial declines have been reported in *Plasmodium*-specific antibody responses to low or undetectable levels within months, and even weeks of a clinical episode after reduced parasite exposure, despite an initial robust response (56, 57, 62–69). Studies modeling the longevity of *P. falciparum* merozoite-specific IgG antibodies have estimated average half-lives of <10 days in children recovering from clinical malaria (67). Similarly, short antibody half-lives ranging from only 2–7 weeks has been reported in asymptomatic children during the dry season in The Gambia (68), which contrasts dramatically with those of antibody responses to viral and bacterial antigens such as vaccinia, measles and tetanus that reportedly persist for decades following a single exposure (70–72). It is possible that antibody responses measured following a clinical malaria episode may reflect the output of short-lived antibody-secreting cells, which typically produce an immediate but transient wave of antibodies to control infection. In line with this idea, antibody-secreting cells have been detected transiently in Ugandan children immediately following acute malaria, and were found to proportionally increase again upon a second clinical episode (73). Modeling studies extended these observations, estimating the longevity of both short- and long-lived antibody-secreting cells in African children to range from 2–10 days, and 3–9 years, respectively (74). Thus, together these findings suggest that a long-lived humoral response to malaria infection can potentially be sustained after decay of transient antibody-secreting cells. More detailed mechanistic investigations are much needed to determine how parasite-specific antibody responses are sustained over time and the factors that modulate the generation and maintenance of antibody-secreting cells to infection.

## The Acquisition of Memory B Cells to Natural Malaria Infection

Several studies have now shown the induction of *Plasmodium*-specific MBCs in response to malaria infection; although individuals exposed to high seasonal transmission have been reported to induce only low frequencies of MBCs or to lack detectable MBCs, even after exposure to parasitic loads sufficient and capable of inducing antibody responses (57, 75, 76). Consistent with the slow acquisition of antibody responses in endemic settings, the prevalence and breadth of *Plasmodium*-specific MBCs appear to develop incrementally with age and exposure (57, 59, 77). Longitudinal studies of children and young adults in an area of high seasonal transmission in Mali demonstrated a delayed, age-associated development of MBCs specific for *P. falciparum* merozoite antigens despite repeated infections annually (57). Moreover, the prevalence of MBCs acquired by children following acute malaria appeared to diminish substantially within the 6-month dry season, contrasting with the stable frequency of MBCs to tetanus toxoid vaccination in the same subjects (57). While seasonal transmission prevented the longevity of these cells to be determined beyond the dry season, this finding implies an impaired maintenance or generation of MBCs in children exposed to high transmission as they acquire clinical protection.

In contrast, individuals residing in areas of low transmission or subjected to fewer clinical episodes have been shown to generate stable *Plasmodium*-specific MBCs without frequent boosting (77–85). The frequencies of *P. falciparum*-specific MBCs detected in Thai adults that had experienced limited episodes reportedly remained stable over time, with an estimated half-life of approximately 7.5 years (81). Similarly, malaria-specific MBCs have been described to be well-maintained in individuals with a history of acute malaria but have since lived in the absence of persistent infection (83, 84). In parallel, low levels of exposure can effectively sustain parasite-specific antibody responses (79, 82, 84, 86, 87), with antibody half-lives estimated to be substantially longer than that of highly-exposed individuals, ranging from months to years (32, 81, 88), suggesting sustained antibody production from long-lived plasma cells. Collectively, these findings reveal that MBCs can be generated and sustained following a limited number of clinical episodes, while repeated infections in endemic areas could have a detrimental effect on the generation of B cell memory.

The characterization of malaria-specific MBCs to date has relied predominantly on *in vitro* stimulation and differentiation of circulating MBCs into antibody-secreting cells followed by detection of antigen-specific clones by ELISPOT assays. This approach precludes phenotypic analysis of the malaria-specific MBC compartment. More detailed investigations are needed to determine the ontogeny of detected MBCs and whether they contribute to effective clinical immunity in malaria-exposed individuals. Whereas, the induction of IgG-expressing MBCs has been the primary focus over the past several years, a few studies have identified IgM MBCs in malaria-exposed individuals and in malaria mouse infection models (59, 89, 90), with those induced by murine malaria found to rapidly differentiate into plasmablasts upon antigenic restimulation (89). Interestingly, MBCs isolated from malaria-exposed individuals have been described to have undergone Ig somatic hypermutations (76), and accumulate further mutations upon acute malaria, with IgM being the dominant isotype expressed prior to re-infection (59).

The use of murine infection models has also provided insight into the development of B cell memory to malaria. Although murine infection does not mirror all the features of human malaria, there are genetic and phenotypic parallels between the human parasite and rodent counterparts (91, 92). A few studies have detected the development of IgG memory B cells following non-lethal *P. chabaudi* infection (89, 90, 93, 94), associated with efficient generation of secondary GCs and enhanced control of re-infection (90, 95). In contrast, *P. yoelii* blood-stage infection has been suggested to ablate vaccination-induced MBCs (96), and further reduces the development of mature, isotype-switched MBCs against pre-erythrocytic parasite antigen, which was associated with the induction of pro-inflammatory cytokines and chemokines that may hinder effective T and B cell interactions (97).

## Immunological Processes Modulating the Induction of Memory B Cells in Malaria

T follicular helper (Tfh) cells are a crucial subset of CD4^+^ T cells that orchestrate B cell memory development by providing crucial survival and differentiation signals to B cells during the GC response (98), and have been shown to be important for the control of *Plasmodium* infection (99–105). The development of Tfh cells has not been extensively investigated in human malaria infection, however, a recent study identified the induction of T helper 1 (Th1) cell-like CXCR3^+^PD-1^+^ Tfh cells in the circulation of Malian children following acute *P. falciparum* malaria (106). Circulating Tfh cells have been proposed to be a surrogate measure of GC-derived Tfh cell responses to human infection (107, 108) and thus constitutes a valuable proxy to investigate Tfh cell biology in human settings. Circulating Tfh cells in malaria-exposed children were shown to express the Th1 lineage-defining transcription factor T-bet, and had limited functional capacity to support MBC responses *in vitro* (106). CXCR3^+^PD-1^+^ Tfh cells have also been identified in Brazilian adults during acute *P. vivax* malaria (109). Interestingly, their proportions positively correlated with repeated malaria episodes (109), implying that circulating Tfh cells accumulate with sustained parasite exposure. However, whether or not the detected Tfh cells facilitate the induction of MBCs that confer protection to disease over time remains to be investigated in larger cohorts and different transmission settings. In addition, circulating follicular regulatory T (Tfr) cells have been postulated to interfere with Tfh cell responses to infection (110). Tfr cells expressing the inhibitory molecule CTLA-4, have been detected at increased frequencies after acute malaria in Malian children (110). Future studies will be important in determining if the transition between susceptibility to clinical disease and asymptomatic infection is associated with the development of functional Tfh cell responses that support the induction of B cell memory populations.

Similar to human malaria, CD4^+^ T cells induced by murine infection have been described to express a dual Th1- and Tfh-associated phenotype, including chemokine receptor CXCR5, and cytokines IL-21 and IFN-γ (99, 111, 112). Precursor Tfh cells induced in response to *P. berghei* severe malaria infection also display a Th1 cell-like phenotype, characterized by the upregulation the chemokine receptor CXCR3 and transcription factor T-bet (113). Moreover, the expression of T-bet impaired the differentiation of Tfh cell precursors into mature Tfh cells, which significantly suppressed the development of GC B cells, plasma cells and MBCs (113). Notably, genetic ablation of T-bet or neutralization of the pro-inflammatory cytokines interferon (IFN)-γ and tumor necrosis factor (TNF) promoted Tfh cell differentiation, and restored previously impaired GC responses, demonstrating that inflammatory responses associated with the induction of symptomatic malaria reduce the magnitude of MBC responses by modulating effective Tfh cell development (114). In support of this concept, exogenous IFN-γ has been shown to reduce Tfh and GC B cell responses to *Plasmodium yoelii* (102), and the blockade of CXCR3-mediated signals was found to enhance precursor Tfh cell accumulation in the spleen of malaria-infected mice, thereby favoring parasitic clearance (115). Other inflammatory pathways such as type I IFN signals have also been recognized to indirectly limit Tfh and GC B cell responses to infection (116, 117), although the precise mechanisms by which this occurs is not yet clear. Recent studies suggest that the damage-signal sensor P2X7 (118), IFN regulatory factor 3 (119), and changes in metabolism and proliferation, as well as altered gene expression of chemokine receptors (120) influence the bifurcation of Tfh and Th1 cell differentiation during murine infection. Whereas, inflammatory responses to malaria appear to dampen the magnitude of the MBC response to infection (114), effective control of blood-stage malaria and its associated pro-inflammatory responses appears to permits normal progression of Tfh cell development (113, 121), giving rise to T cell memory that responds to secondary infection (112).

Whereas, it is reasonable to assume that the aforementioned modulation of Tfh cell function by inflammatory pathways elicited in response to infection is responsible for the reduced B cell responses observed during acute infection, a direct effect of IFN-γ-mediated signaling or intrinsic expression of T-bet in B cells cannot be ruled out. *P. yoelii* infection has recently been shown to induce a subset of B cells to express the transcription factor T-bet, and deletion of B cell-specific IFN-γ receptor or T-bet deletion elevated antibody responses to infection (122, 123). Further work to establish if inflammation directly modulates the differentiation and functional capacity of B cells is needed to address this question.

## Atypical Memory B Cells: Friends or Foes?

There is evidence that *Plasmodium* parasites can directly modulate B cell function. *In vitro* studies have shown that the parasite can directly activate human naive B cells via a cysteine rich inter-domain region of *P. falciparum* erythrocyte membrane protein 1 (124, 125) and downstream toll-like receptor signaling (126), which may lead to non-specific polyclonal activation of B cell responses. CD27-expressing B cells were observed to be the major responding population (124, 125), suggesting a potential impact on the MBC compartment. In line with this idea, acute *P. falciparum* infection has been reported to modulate systemic mediators of B cell activation and survival (127, 128), which has been associated with an early proliferation of MBC subsets, prior to the induction of GC responses following experimental human infection (128), supporting a potential role of bystander activation of non-specific MBC subsets that enables parasite evasion.

Insight into the notion that B cell memory induction in malaria may be dysregulated surfaced upon the characterization of an expansion of MBCs exhibiting an atypical phenotype following persistent malaria exposure (17, 84, 129, 130). Originally described as an exhausted subset of MBCs implicated in humoral deficiencies in HIV infection (131), a phenotypically similar CD27^−^CD21^−^ circulating MBC subset notable for their expression of inhibitory receptors, was detected in *Plasmodium*-infected individuals following acute malaria in Brazil (109), Gabon (132), Ghana (85), Gambia (77), India (133), Kenya (84, 130), Mali (15, 17, 129, 134, 135), Papua New Guinea (136, 137), Thailand (78), and Uganda (16, 73, 138), and further exacerbated by HIV co-infection (139, 140). Studies to date corroborate that the accumulation of this subset is influenced by the level of parasite exposure. Higher frequencies of atypical MBCs were found in adults compared to children with shorter exposure histories (17). Similarly, Malian adults from high transmission settings were found to have higher frequencies of these cells than Peruvian individuals residing in a low transmission area (129). A decline in the atypical MBC pool was observed during 12 months without malaria transmission (84), further substantiating a role of persistent parasite exposure in the maintenance of this population.

Recent work has investigated the function of these atypical MBCs and their relationship to classical MBCs. Evaluation of the variable gene repertoires of atypical MBCs proposed that they share a common developmental precursor to classical MBCs (15, 132, 141), however, emerging work support that atypical MBCs are phenotypically and functionally different to classical MBCs. There is still conflicting evidence on the effector capacity of atypical MBCs from malaria-infected individuals. Whereas, some support the notion that atypical MBCs are capable of secreting neutralizing antibodies (132), subsequent work contend their lack of active antibody secretion (15, 16). Atypical MBCs have been reported to express lower levels of surface IgG (136), possess reduced B cell receptor signaling, as well as impaired interleukin (IL)-6, IL-8 and antibody production *in vitro* (15, 16). Atypical MBC frequencies in malaria-exposed individuals have been shown to be positively correlated with pro-inflammatory cytokine levels such as IL-8 (136), and their expression of the inhibitory molecule FcRL5 has been proposed to be a marker of dysfunction associated with increased malaria exposure (16). FcRL5-expressing atypical-like MBCs in healthy individuals have been distinguished by their higher expression of inhibitory receptors such as PD-1 and transcription factors *Tbx21, Bcl-6*, and *Sox5* and blunted proliferation capacity compared to FcRL5^−^ B cells (142). In line with these findings, recent studies revealed that atypical MBCs in Malian children express *Tbx21* (134), which encodes the Th1 transcription factor T-bet (143). Emerging evidence that T-bet becomes up-regulated in various disease contexts in subsets of B cells that share many features in common with atypical MBCs found in malaria-infected individuals. T-bet-expressing B cells have been associated with both protective and pathogenic roles depending on specific settings, which include bacterial (21), parasitic (144–146) and viral infection (147–153), cancer (154–156), autoimmune conditions (157–164), and aging (165). In malaria, atypical MBCs expressing high levels of T-bet have also been found to express decreased levels of the co-stimulatory molecule CD40 and reduced phosphorylation of B cell receptor signaling molecules (134), suggesting that T-bet expression might reduce B cell effector function. In line with this concept, the frequency of malaria episodes was found to be associated with increased T-bet^hi^ MBC in a small group of children (134). On the other hand, longitudinal cohort studies have reported that low parasite density malaria is associated with persistence (78) or accumulation of atypical MBCs over time, which raises the possibility that atypical MBCs contribute to developing immunity (73, 138), although T-bet expression was not examined in these cells. Furthermore, similar atypical MBCs that express FcRL5 have been recently detected in *P. chabaudi*-infected mice (166, 167), but appear to display normal features of proliferation and Ig expression, representing a short-lived population of activated MBCs (167). Collectively, a causal relationship between atypical MBCs and immune protection or disease progression remains unclear, and more studies are urgently needed to determine the functional significance of atypical MBC expansion in the acquisition of humoral immunity to malaria and the precise contribution of the transcription factor T-bet in shaping atypical MBC function.

## Concluding Remarks and Future Perspectives

Emerging evidence both in human field studies and murine infection models are beginning to address how the induction of humoral immune responses is compromised during acute malaria, and the consequences of these processes for the establishment of long-term immunological B cell memory. There is now considerable evidence that exposure to *Plasmodium* parasites is associated with altered proportions of circulating memory B cells, including an expansion of atypical memory B cells. Further studies to unravel the complexity of this diverse memory B cell compartment, in terms of origin, function and protective capacity of different subpopulations are urgently needed. Information about the level of homology between human and mouse memory B cell subsets is lacking, particularly in relation to their surface marker expression patterns, which makes it difficult to infer how findings in malaria infection models translate into human settings. Gene expression analysis studies, including how transcription factors and the cytokine environment influence these processes might be required to overcome these issues and establish functional correlations between human studies and much needed mechanistic work in mice.

Given the critical importance of antibodies and long-lived humoral memory in immunity to malaria, an in-depth understanding of the factors that delay their development is undoubtedly required to inform the design of targeted therapeutic strategies to enhance immune responses to the parasite and protect against disease susceptibility. Detailed characterization of the immune processes by which B cell memory to malaria is generated and the specific effector populations required to confer protection, will undoubtedly benefit vaccine development and optimisation efforts, especially in light of the modest efficacy levels achieved to date with current vaccination regimes at a population level (168, 169).

## Author Contributions

All authors listed have made a substantial, direct and intellectual contribution to the work, and approved it for publication.

### Conflict of Interest Statement

The authors declare that the research was conducted in the absence of any commercial or financial relationships that could be construed as a potential conflict of interest.
